# Free Induction Decay Optically Pumped Magnetometers: A Review

**DOI:** 10.3390/s26196103

**Published:** 2026-09-26

**Authors:** Jiarui Liu, Xiaodong Liu, Yuantian Lu, Mingjin Zhuang, Xiaojun Liu, Wanhua Zhu, Tian Zhao

**Affiliations:** 1 Aerospace Information Research Institute, Chinese Academy of Sciences, No. 9 Dengzhuang South Road, Beijing 100094, China; liujiarui22@mails.ucas.ac.cn (J.L.);; 2 School of Electronic, Electrical and Communication Engineering, University of the Chinese Academy of Sciences, Beijing 100049, China

**Keywords:** atomic magnetometers, geomagnetic applications, free induction decay

## Abstract

Free induction decay (FID) optically pumped magnetometers are among the most sensitive quantum sensors available for high-precision magnetic field measurements. This review summarizes recent advances in FID magnetometers, which represent a critical technological route that suppresses systematic errors by temporally separating atomic polarization from magnetic field detection. This review covers light-modulated optical pumping schemes (such as the Bell–Bloom mode), radio-frequency excitation schemes, and magnetic-field-enhanced pumping schemes. Key technological developments, including chip-scale MEMS fabrication, optical multipass architectures, advanced frequency estimation algorithms, and error suppression methods, are also discussed. Finally, emerging trends toward higher integration, environmental robustness, and intelligent signal demodulation are highlighted. These trends provide insights into the development of next-generation high-performance quantum sensors and their growing role in biomedical and geophysical applications, as well as in fundamental scientific research.

## 1. Introduction

Optically pumped magnetometers (OPMs), which rely on the interaction between atomic spins and polarized laser light, have achieved high sensitivities approaching the level of superconducting quantum interference devices (SQUIDs) without the need for cryogenic cooling [1,2,3]. This capability provides a balance between measurement performance and device portability [4,5,6]. OPM technology has demonstrated broad prospects across numerous scientific and practical applications. In geomagnetic monitoring and navigation, it provides valuable support for mineral exploration, geomagnetic background analysis, and magnetic characterization in complex urban environments [7,8,9]. In the biomedical sector, its applications have driven advancements in wearable magnetoencephalography (MEG) and magnetocardiography (MCG). These advancements enhance the spatiotemporal resolution and signal quality of non-invasive neural and cardiac activity detection [10,11,12]. Furthermore, the technology shows growing potential for fundamental physics research, including the search for new physical phenomena and dark matter. It also holds significant promise in defense and security, particularly for aerial magnetic anomaly detection (MAD) [13]. 

Continuous-wave (CW) and pulsed free-induction-decay (FID) magnetometers represent two different measurement paradigms. In CW operation, the optical pumping and readout processes are maintained continuously or quasi-continuously. This mode provides a real-time output without a preparation dead time, offering a high continuous update rate and relatively simple steady-state signal processing. However, the continuous presence of the pump light during measurement may introduce pump-induced light shifts, optical power broadening, and additional systematic effects [14]. In unshielded Earth-field environments, CW systems may also exhibit orientation-dependent heading errors and nonlinearities associated with the coupled light–atom dynamics.

In FID operation, spin preparation or excitation is followed by a defined free-evolution interval. Depending on the specific architecture, the pump or excitation drive may be removed, strongly attenuated, or otherwise arranged to minimize its influence on the free-precession dynamics [14]. In architectures where the pump field is removed or strongly attenuated during this interval, the temporal separation can substantially reduce pump-induced effects such as light shifts and power broadening. The observed linewidth then more closely reflects the transverse relaxation dynamics [15]. The transient FID waveform can also provide information beyond the central precession frequency, including the relaxation time, the initial phase, and, when spectrally resolvable, multiple hyperfine or Zeeman components [16,17]. These features can improve the interpretation of systematic effects and support high-accuracy magnetic-field estimation. FID operation nevertheless involves significant trade-offs. The preparation and detection stages introduce dead time and limit the repetition rate of independent field estimates. Consequently, the effective sampling and dynamic-tracking bandwidth may be lower than that of a continuously interrogated sensor. The FID amplitude also decays during the observation window, so accurate frequency estimation requires careful coordination of the measurement timing with the signal processing and the preparation and excitation sequence. In addition, probe-induced perturbations may remain relevant for resonant absorptive readout, even when the pump field is absent. In summary, FID and CW magnetometry should be considered complementary rather than competing measurement schemes. The choice between FID and CW schemes represents a trade-off among continuous operation, sensitivity, measurement bandwidth, systematic-error suppression, and signal-processing complexity.

To identify relevant studies, we searched Google Scholar, IEEE Xplore, and arXiv for literature published from 1 January 2015 to 30 June 2026. The final search was completed on 20 July 2026, and selected foundational publications dating back to 1961 were included. The main search terms included “free induction decay magnetometer”, “FID OPM”, “free precession magnetometer”, “pulsed atomic magnetometer”, “optical pumping magnetometer”, and “spin precession”, together with their close variants and relevant combinations. Retrieved records were screened for relevance, and the full texts of potentially relevant studies were assessed. Studies were included when they addressed the generation, detection, modeling, calibration, or application of transient free precession or FID signals for magnetic field measurement. Studies without an FID component and studies unrelated to magnetic field sensing were excluded from the main architectural comparison. The search focused on the physical principles, excitation architectures, time domain signal processing methods, error suppression strategies, and the application landscape of FID magnetometers.

In this review, an FID magnetometer is defined operationally as a system in which spin preparation or excitation is temporally separated from signal readout, and the magnetic field information is extracted from the transient free precession of the spin ensemble during a subsequent free-evolution interval. This definition distinguishes FID magnetometers from related optically pumped magnetometers. Pulsed OPMs that maintain a driving field during detection, continuous-wave spin-exchange relaxation-free (SERF) magnetometers, and Pound–Drever–Hall (PDH) systems used only for optical frequency locking are not treated as true FID systems. Studies involving SERF, PDH, or other related OPM techniques are included only when an FID transient response is explicitly measured, used for calibration, or combined with another operating mode. Such studies are identified as examples of application scenarios.

This review aims to cover the technical landscape of FID-OPMs, including their physical principles, hardware architectures, signal processing algorithms, and applications. The paper is organized as follows: Section 2 introduces the physical foundations using Bloch equations and analyzes noise sources and sensitivity limits. Section 3 classifies FID magnetometer architectures, covering synchronous modulation (Bell–Bloom), radio-frequency (RF) excitation, and magnetic-field-enhanced pumping. Section 4 highlights hardware optimization and signal processing, covering chip-scale microelectromechanical system (MEMS) vapor cell fabrication, multipass cell technologies, and algorithms such as singular value decomposition (SVD) filtering and compressive sensing. Section 5 discusses error suppression and environmental adaptability, including heading error calibration, dead-zone elimination, and the nonlinear Zeeman effect. Section 6 summarizes applications in biomagnetic imaging, geomagnetic monitoring, industrial non-destructive testing (NDT), and fundamental physics. Section 7 identifies current technical bottlenecks and future trends in integration, dynamic environmental adaptation, and intelligent demodulation. Section 8 concludes with the key findings and the significance of FID technology in quantum sensing.

## 2. Fundamental Physics of FID

### 2.1. Mechanisms of Atomic Spin Coherence and Free Precession

The underlying physical principle of FID magnetometers is based on the preparation, evolution, and optical readout of spin-coherent states in an atomic ensemble (such as alkali metal atoms or metastable ^4^He atoms) subjected to an external magnetic field. From a quantum mechanical perspective, atoms undergo Zeeman splitting in the presence of an external magnetic field ($B_{0}$), where the interaction between the atomic magnetic moments and the magnetic field induces shifts in the energy levels. Macroscopically, these dynamics can be characterized by the phenomenological Bloch equations. Equation (1) describes the free-evolution stage after the preparation or tipping process:

(1) $$\frac{dM}{dt}=\gamma M\times B_{0}-\frac{M_{⊥}}{T_{2}}-\frac{M_{∥}-M_{0}}{T_{1}}$$

where γ is the gyromagnetic ratio, $B_{0}$ is the external magnetic field, and M is the ensemble magnetization. $M_{⊥}$ and $M_{∥}$ are the transverse and longitudinal components of the magnetization vector, respectively. $T_{2}$ and $T_{1}$ represent the transverse and longitudinal relaxation times, which govern the decay of spin coherence and the recovery of population differences. Finally, $M_{0}$ denotes the steady-state longitudinal magnetization established by the optical pumping.

Figure 1 illustrates the basic operating principle of the FID magnetometer. A laser pumps the atomic ensemble to generate spin polarization through the light–atom interaction. An FID signal is then induced by, for example, modulating the light intensity, and its frequency information is used for magnetic field measurement. The operational cycle of an FID magnetometer can be divided into two stages: spin preparation and signal detection. During the spin-preparation stage, optical pumping transfers angular momentum from resonant photons to the atomic ensemble, producing a highly polarized spin state. The method used to initiate precession depends on the magnetometer architecture. In RF-excited schemes, a short RF magnetic pulse rotates the magnetization away from its equilibrium direction. In all-optical schemes, synchronous modulation or pulsed optical pumping drives the atoms into a non-equilibrium state and initiates coherent spin precession without RF excitation [18].

During the subsequent detection stage, the atomic spin ensemble enters free evolution and produces an FID signal. The signal precesses around the ambient magnetic field ${\vec{B}}_{0}$ at the characteristic Larmor frequency (*f*_L_).

(2) $$\begin{array}{c} f_{L}=\frac{\gamma |{\vec{B}}_{0}|}{2\pi } \end{array}$$

To monitor the precessing atomic magnetization, optical probing is commonly used to detect the modulation of a transmitted probe laser beam by the spin-polarized atomic vapor. Depending on the probe laser detuning, optical readout is primarily implemented through off-resonant dispersive detection or resonant absorptive detection. Figure 2 shows a representative FID experimental setup that integrates excitation and dispersive detection. In dispersive detection, spin-dependent circular birefringence produces periodic polarization rotation through the optical Faraday effect, with low photon absorption and thus reduced perturbation to the atomic coherence. In absorptive detection, spin-dependent circular dichroism produces periodic modulation of the transmitted optical intensity, providing a larger signal with a simpler optical configuration. The choice between these approaches depends mainly on the trade-off between optical perturbation, signal strength, and optical complexity. For both optical detection modalities, the acquired time-domain FID signal S(t) during free precession can be modeled as a damped sinusoidal oscillation. In the regime where nonlinear Zeeman effects are suppressed or when the detection targets the dominant transition, the FID signal is accurately described by a single exponentially damped sinusoid:

(3) $$\begin{array}{c} S(t)=A\cdot \exp\left(-\frac{t}{T_{2}}\right)\cdot \cos(2\pi f_{L}t+\phi )+n(t) \end{array}$$

where *A* is the signal amplitude, *T*_2_ is the transverse relaxation time, ϕ is the signal phase, and n(t) denotes signal noise.

This two-stage cyclic operation largely separates the frequency-measurement process from the polarization process in the time domain. It thereby provides a foundation for high-sensitivity measurements in FID magnetometers.

### 2.2. Noise Analysis and Sensitivity Assessment

The sensitivity of a magnetometer is defined as the minimum detectable magnetic field variation per unit bandwidth, expressed in pT/√Hz or fT/√Hz. The sensitivity of an FID magnetometer depends fundamentally on the signal-to-noise ratio (SNR), the temporal parameters of the operating cycle, and the frequency estimator used. By employing the Cramér–Rao lower bound (CRLB) for parameter estimation of exponentially damped sinusoids, the fundamental sensitivity limit can be formulated as [21]

(4) $$\delta B_{CRLB}=\frac{1}{\gamma A}\sqrt{\frac{12\rho_{n}^{2}}{T_{m}^{3}}}\cdot \frac{1}{\sqrt{\eta }}\cdot C_{est}(T_{m},T_{2})$$

where $\rho_{n}$ is the noise spectral amplitude density, $T_{m}$ is the single-shot observation time, and $\eta =T_{m}/T_{c}$ is the duty cycle accounting for the total cycle duration $T_{c}$ (which includes the dead time during polarization). $C_{est}(T_{m},T_{2})$ is an estimator-dependent dimensionless correction factor that accounts for the continuous decay of the SNR during the observation window.

As indicated by the CRLB model, the transverse relaxation time $T_{2}$ influences the usable observation window and the SNR decay rate. The total transverse relaxation rate is collectively governed by several intrinsic and extrinsic mechanisms:

(5) $$\frac{1}{T_{2}}=\frac{1}{T_{SE}}+\frac{1}{T_{SD}}+\frac{1}{T_{buff}}+\frac{1}{T_{wall}}+\Gamma_{\nabla B}$$

where $1/T_{SE}$ and $1/T_{SD}$ denote the spin-exchange and spin-destruction relaxation rates between alkali atoms, respectively. The extrinsic contributions from buffer gas collisions, cell walls, and field gradients are explicitly defined as

(6) $$\frac{1}{T_{buff}}=N_{bg}\overset{\text{-}}{v}\sigma_{bg}$$

(7) $$\frac{1}{T_{wall}}=\frac{D}{\Lambda^{2}}$$

(8) $$\Gamma_{\nabla B}=\frac{\gamma^{2}|\nabla B{|}^{2}R^{4}}{175D}$$

where $N_{bg}$, $\overset{\text{-}}{v}$, and $\sigma_{bg}$ represent the buffer gas number density, the mean relative thermal velocity, and the alkali–buffer collision cross-section, respectively. The wall-collision rate $1/T_{wall}$ is constrained by spatial diffusion across the characteristic diffusion length Λ of the vapor cell geometry, governed by the diffusion coefficient D, where D∝1/P, with P being the buffer gas pressure. Finally, $\Gamma_{\nabla B}$ accounts for dephasing induced by spatial magnetic field gradients ∇B within a cell of radius R in the motional narrowing regime [16]. Prolonging $T_{2}$ via optimized buffer gas pressure and spatial gradient compensation directly suppresses linewidth broadening, thereby enabling the sensor to approach its theoretical sensitivity limit [22,23,24].

In this optimization process, computational modeling and numerical exploration play an important role during the pre-experimental design phase. Numerical simulations based on spatial diffusion formalisms and finite-element analysis can be used to model atomic wall-collision dynamics and evaluate suitable buffer gas pressures across diverse vapor cell geometries. Concurrently, electromagnetic modeling enables the calculation of spatial magnetic field gradients ∇B and assists in the structural design of compensation coils and magnetic shields to suppress gradient-induced dephasing $\Gamma_{\nabla B}$. Furthermore, by employing numerical solvers and parameter grid searches based on the time-dependent Bloch equations or density matrix formalisms, researchers can systematically simulate transient spin evolution across multi-dimensional parameter spaces (such as optical detunings, pumping durations and RF tipping angles) prior to physical fabrication. This pre-fabrication modeling assists in identifying favorable observation times and duty cycles, and it helps assess the achievable estimation performance relative to theoretical limits.

Noise in FID-OPMs can be classified into three categories. First, spin projection noise arises from the quantum uncertainty of spin measurements; for an ensemble of N atoms, the absolute spin-variance fluctuations scale as √N, so that the corresponding magnetic-field-equivalent sensitivity floor improves as 1/√N [25,26]. Second, photon shot noise originates from the Poissonian statistics of photon arrival at the detector and scales with the square root of the probe laser power [27]. Third, technical noise includes laser power and frequency fluctuations, electronic noise, temperature variations, and residual magnetic field fluctuations. Many of these contributions can be suppressed through design choices such as differential gradiometer architectures [21]. For FID signals with exponentially decaying amplitude, the frequency estimation precision is further influenced by the single-shot sampling duration and sampling rate. Through modern digital signal processing techniques (detailed in Section 4), the system can approach the theoretical sensitivity limit. For context, pulsed or FID-related magnetometers operated under shielded low-field or SERF-like conditions have reported sensitivities at the femtotesla level [28]. 

More generally, reported sensitivity values should be interpreted in the context of the corresponding operating conditions. Measurements performed in shielded zero or low field environments are not directly comparable to scalar measurements conducted in unshielded earth field backgrounds. Comparisons should also account for the sensing configuration, sensing volume, cell geometry, bandwidth or cycle rate, and averaging time, since these factors affect the noise limits and reported sensitivity. Therefore, the values are intended to illustrate representative capabilities and design trade-offs rather than establish a universal ranking. 

Furthermore, the dynamic performance of an FID magnetometer involves several distinct frequency-related parameters. The FID cycle rate $f_{c}$ is the repetition frequency of the complete preparation and detection sequence and determines the update rate of the magnetic field estimate. If one independent estimate is obtained per cycle with approximately uniform sampling, the corresponding Nyquist frequency is $f_{Nyq}\approx f_{c}/2$. This sampling limit is distinct from the spectral linewidth of an individual FID waveform, which for a single exponentially decaying component is approximately $\Delta \nu_{FWHM}\approx 1/(\pi T_{2})$ for a Lorentzian line shape. The actual estimator bandwidth additionally depends on the observation window, signal-processing algorithm, signal-to-noise ratio, and processing latency, while the closed-loop tracking bandwidth also includes the controller, actuator, and loop delays. Thus, cycle rate, spectral linewidth, estimator bandwidth, and closed-loop bandwidth should not be treated as interchangeable quantities.

## 3. Classification of FID Magnetometers

In FID magnetometers, a key physical distinction lies in the method used to tip the atomic magnetization and initiate spin precession, which has a significant influence on the overall performance and design of the magnetometer. As illustrated in Figure 3, FID magnetometers are categorized into three primary architectures based on their precession initiation mechanism: light-modulated optical pumping, radio-frequency excitation, and magnetic-field-enhanced optical pumping. While these architectures represent distinct excitation routes widely used for comparative performance evaluations in the literature, they also couple to various spin preparation schemes, optical readout configurations, and scalar or vector measurement modalities. The physical mechanisms and typical implementations of these three classes are detailed below.

### 3.1. Light-Modulated Optical Pumping Schemes

Light-modulated optical pumping schemes utilize periodic modulation of laser parameters, such as intensity or frequency, to prepare atomic spin coherence without the application of an external radio-frequency tipping pulse. In a Bell–Bloom configuration, the optical modulation frequency (*f*_mod_) is applied during the preparation stage and is synchronized with the atomic Larmor frequency (*f*_L_). This modulation produces a driven and phase-locked spin response that progressively builds the transverse spin polarization [25,28]. The driven response during this stage is not itself free precession.

After the desired spin coherence has been established, the optical modulation train is either terminated or detuned sufficiently to remove the coherent optical driving effect. The FID detection interval begins at this point. During this interval, the atomic ensemble evolves in the background magnetic field without the Bell–Bloom excitation drive, and the resulting transient signal is regarded as the free-induction-decay response.

(9) $$\begin{array}{c} f_{mod}=\frac{1}{T_{mod}}\approx f_{L}=\frac{\gamma |{\vec{B}}_{0}|}{2\pi } \end{array}$$

Depending on the modulation method, these schemes can be categorized as amplitude modulation (AM) or frequency modulation (FM). In AM schemes, the pump laser is periodically switched on and off or modulated in intensity. When the modulation frequency matches the Larmor frequency, the atomic precession remains phase-locked to the optical pulses, and macroscopic spin coherence builds up progressively over multiple modulation cycles [25,29]. In contrast, FM schemes maintain a constant optical power while periodically tuning the laser frequency between resonant and off-resonant regions. Compared with the AM mode, the FM mode utilizes laser power more efficiently and further suppresses errors associated with light shifts [25].

Yoon et al. proposed an all-optical single-beam FID magnetometer scheme based on laser mode hopping [30]. As shown in Figure 4, the system employs FM and achieves a sensitivity of 3.77 pT/√Hz in Earth-scale fields. The operating principle relies on rapidly modulating the laser driving current to induce abrupt frequency hops between atomic transition lines or longitudinal laser modes. Compared with conventional approaches, this scheme enables high-efficiency optical pumping within a short duration and offers an effective excitation pathway for all-optical pulsed magnetometers. Chi Fang et al. further showed that superimposing magnetic field pulses onto synchronous modulation reduces spin-exchange relaxation by 91.4% and increases resonance signal strength by 74.0% [31].

### 3.2. RF Excitation Schemes

The RF excitation scheme is one of the most direct methods for generating FID signals. The atomic ensemble is first polarized using a CW pumping beam. A short RF magnetic field pulse near the Larmor frequency is then applied, tipping the polarization vector into the transverse plane. Following the pulse, the atomic ensemble undergoes coherent free precession and produces the characteristic FID signal.

(10) $$\begin{array}{c} \omega_{rf}=2\pi f_{rf}\approx \gamma |{\vec{B}}_{0}| \end{array}$$

The performance of RF-excited FID magnetometers depends critically on the precise control of pulse parameters, including the RF frequency, phase, and pulse duration. Figure 5 shows a typical RF-type FID magnetometer experimental setup [32]. In this scheme, the RF excitation frequency is tuned close to the atomic Larmor frequency, producing coherent spin rotations analogous to Rabi oscillations in quantum systems. In practice, a π/2 pulse is typically chosen to maximize the transverse signal amplitude [20,33,34]. Compared with all-optical schemes such as Bell–Bloom, RF excitation is characterized by high excitation efficiency and flexible manipulation. On the one hand, polarization tipping can be accomplished rapidly with minimal sensitivity to laser power fluctuations. On the other hand, complex spin-state control can be realized by phase variation or composite pulse sequences.

Under Earth-scale magnetic field conditions, Junhao Liu et al. demonstrated that combining synchronous RF pulse adjustment with adaptive filtering achieved a sensitivity of 0.47 pT/√Hz in complex interference environments [34]. 

### 3.3. Magnetic-Field-Enhanced Optical Pumping Schemes

Magnetic-field-enhanced optical pumping schemes introduce an auxiliary excitation magnetic field during the pumping stage to reorient the total magnetic field. This allows for the atomic polarization process to be completed under geometrically optimal conditions. The core principle is to align the effective magnetic field during the polarization stage with the propagation direction of the pump laser. This approach achieves high initial longitudinal spin polarization and generates a large-amplitude FID signal upon entering the detection stage. In conventional single-beam pulsed FID schemes, pumping efficiency decreases significantly when there is a large angle between the field under test and the laser axis. The magnetic-field-enhanced pumping scheme applies an auxiliary magnetic field parallel to the laser direction, ensuring that the total field sensed by the atoms during the pumping stage is aligned with the laser axis. Under this condition, the pumping process is decoupled from the depolarization effects caused by Larmor precession, which enables rapid accumulation of the maximum possible magnetic moment [35]. Once pumping is complete, the auxiliary field is rapidly switched off. The polarization vector then undergoes free precession in the ambient magnetic field at a larger tipping angle than in conventional schemes. This method results in an FID signal with a high initial amplitude.

A representative implementation of this scheme is shown in Figure 6, including the auxiliary-field coils and rapid field switching. Research has demonstrated that magnetic-field-enhanced pumping schemes offer significant advantages in specific application scenarios [25,36]. Compared to simple single-pulse schemes, enhanced pumping generates FID signals with a higher initial SNR due to more thorough polarization. Since the polarization process is completed under a parallel field, higher pump laser power can be used without risk of premature dephasing of the precession signal. Furthermore, compared to the Bell–Bloom mode, which requires precise frequency feedback, the magnetic-field-enhanced optical pumping scheme has lower requirements for laser modulation frequency stability and exhibits greater robustness. However, its sensitivity is limited by the switching speed of the auxiliary field and the stability of that field.

Magnetic-field-enhanced optical pumping is often used in conjunction with a dual-beam architecture. In this configuration, one pump beam works with the excitation magnetic field to achieve efficient polarization, while a separate probe beam orthogonally monitors the precession process. By providing both spatial and temporal isolation of the pumping and detection functions, this architecture enhances the absolute accuracy of the measurement and effectively suppresses the systematic biases inherent in conventional single-beam systems [35].

### 3.4. Comparative Summary of FID Magnetometer Architectures

The three excitation architectures discussed above provide different solutions to the problem of initiating transverse spin coherence in an FID magnetometer. Light-modulated optical pumping schemes offer an attractive all-optical implementation with relatively low hardware complexity and no need for a dedicated RF tipping coil. They can therefore be useful for compact or chip-scale sensors. Their performance, however, depends on the stability and timing of optical modulation, and accurate synchronization between the modulation frequency and the Larmor frequency may be required during the preparation stage.

RF-excited schemes provide direct and flexible control of the spin-tipping process. The RF pulse amplitude, phase, duration, and composite-pulse sequence can provide precise control over the initial transverse magnetization and improve the reproducibility of the excitation process. These features are advantageous when rapid excitation, accurate pulse control, or vector manipulation is required. The main costs are the need for an additional RF coil and electronics, increased system complexity, possible electromagnetic interference, and the need to control RF detuning and pulse transients.

Magnetic-field-enhanced optical pumping schemes use an auxiliary magnetic field during the preparation stage to align the effective field with the pump-beam direction. This can improve the initial polarization and thereby increase the FID signal amplitude, particularly when the field under test is not favorably oriented with respect to the optical axis. Such schemes may therefore be useful in mobile, vector, or wide-orientation applications. Their limitations include the need for an auxiliary-field coil, fast and stable field switching, and suppression of switching transients and residual field gradients.

These architectures should not be interpreted as simply forming a hierarchy of performance. Each method involves a different balance among excitation efficiency, optical and electronic complexity, synchronization requirements, signal amplitude, power consumption, orientation robustness, and cycle rate. Thus, the three approaches can be viewed as addressing different practical constraints in FID generation rather than representing a simple progression in performance. The most appropriate architecture depends on the application requirements and operating conditions.

To facilitate a structured comparison of the main FID magnetometer architectures, Table 1 summarizes representative systems based on light-modulated optical pumping, RF excitation, and magnetic-field-enhanced optical pumping. The comparison includes the excitation scheme, operating field regime, vapor-cell dimensions, reported sensitivity, reported bandwidth, cycle rate, measurement accuracy, heading error characteristics, and dead-zone mitigation. These parameters are reported according to the conditions described in the corresponding references. Because the systems operate under different magnetic-field regimes and sensing configurations, the values in Table 1 are intended to illustrate representative design trade-offs rather than to provide a direct quantitative comparison. “N/A” indicates that the corresponding parameter was not reported or is not applicable to the operating regime.

## 4. Key Technological Advances

The atomic vapor cell is central to FID magnetometer performance, directly determining volume, power consumption, and fundamental sensitivity. In recent years, the combination of MEMS technology and optical enhancement architectures has propelled FID magnetometers from laboratory-scale devices toward chip-scale sensors. However, miniaturization shortens atomic coherence times and attenuates signal intensity, which poses a severe challenge for precise frequency information extraction from constrained time-domain waveforms. Consequently, high-performance signal demodulation algorithms have become an essential means of compensating for hardware scaling effects and maintaining detection sensitivity.

### 4.1. Micro/Nano-Fabrication of Atomic Vapor Cells

Chip-scale atomic magnetometers demand highly integrated, mass-producible vapor cells. Modern MEMS fabrication etches millimeter- or sub-millimeter-scale cavities into silicon wafers, which are then filled with alkali metals (e.g., Cs) and buffer gases and sealed via silicon-glass anodic bonding. In FID mode, the primary challenge in MEMS vapor cells is strong wall-collision relaxation in confined spaces. By optimizing buffer gas pressure or introducing anti-relaxation coatings, long-lived FID signals have been observed in miniature cells [37,43,44]. Dominic Hunter et al. reduced the light-shift error of a MEMS-based FID magnetometer to the 0.6 nT level [15]. Using the 1.5 mm thick MEMS cell shown in Figure 7, A. McWilliam et al. demonstrated a scalar sensitivity of 2.5 pT/√Hz in a 50 µT Earth-equivalent magnetic field [37]. 

To further enhance sensitivity, optical multipass cell (MPC) technology is widely adopted. This technique utilizes high-reflectivity mirrors or total internal reflection to multiply the effective probe path length through the vapor. It significantly amplifies the FID signal and improves the SNR [32]. Shuguang Li et al. demonstrated that, combined with specific pulse timing control, an FID magnetometer based on a multipass cell can achieve a sampling rate of up to 2 kHz and a sensitivity of 0.7 pT/√Hz in Earth-scale fields [38]. In large-scale dual-chamber RF magnetometers, MPC technology provides the foundation for long-distance, highly synchronized magnetic field measurements [45].

The trade-off between volume and performance governs sensor design. Operating under low-field conditions (<10 μT), Vladislav Gerginov et al. showed that millimeter-scale FID magnetometers can achieve sub-100 fT/√Hz sensitivities through optimized atomic dynamics and signal processing [39]. This provides a valuable technical foundation for chip-scale atomic magnetometers to enter the markets of high-precision geophysical surveys and biomagnetic imaging.

Leveraging the flexibility of MEMS manufacturing, miniature coils or specialized microstructures can be integrated into the same physical unit to enable vector magnetic field measurements. By applying controlled bias fields or microwave drive fields, chip-scale FID magnetometers are able to accurately resolve three-dimensional magnetic field vectors. Using this approach, the setup by Pengbo Jiang et al. achieved a total field sensitivity of 30 pT/√Hz [46]. Such integrated schemes not only reduce sensor size but also further decrease environmental electromagnetic interference by shortening signal transmission paths.

### 4.2. Advanced Frequency Estimation Algorithms

The FID signal can be modeled as an exponentially decaying sinusoidal waveform corrupted by noise. Since the signal duration is limited by the transverse relaxation time, the frequency resolution achievable with conventional fast Fourier transform (FFT) analysis [25] is severely constrained by the length of the time-domain window. To overcome this bottleneck, a series of targeted demodulation and enhancement algorithms have been developed. Although the Hilbert transform [36] is frequently used to extract analytic signals and calculate instantaneous frequencies, it is particularly sensitive to high-frequency noise when processing data with a low SNR, which makes it difficult to meet the requirements for high-precision measurement. Consequently, modern matrix decomposition and subspace denoising techniques, such as backward singular value decomposition (BSVD), have gradually become a research focus for more effective feature extraction and interference suppression in strong noise environments [47].

For scenarios requiring rapid imaging, such as MCG, Mingzhu Bai et al. developed a fast BSVD algorithm that enables efficient reconstruction of weak biomagnetic signals from pulsed OPM data, reducing the recording time from 3.6 ms to 0.6 ms [48]. As illustrated in Figure 8, damaged signals can be restored in the time domain through waveform reconstruction techniques. Dominic Hunter et al. successfully reconstructed a 100 pT oscillating signal using signal averaging and achieved a sensitivity of 3.9 pT/√Hz [49]. 

To address aliasing and the limited resolvable frequency range associated with finite spin coherence, compressive sensing (CS) technology has also been explored for FID magnetometers. CS can exploit prior knowledge of spectral sparsity to reconstruct selected signal components from undersampled measurements. However, its reconstruction capability depends on assumptions about the number of frequency components, the sparsity basis or dictionary, the sampling pattern, and the accuracy of the adopted signal model. When these conditions are satisfied, optimization-based reconstruction may help disambiguate aliased spectral components and increase the practically resolvable frequency range. This apparent extension does not represent an increase in the physical coherence time or the creation of new information beyond what is contained in the measured FID signal. Ruiqi Wang et al. applied this method to FID magnetometers, reporting an apparent extension of the practically resolvable frequency range to 3000 Hz under the assumed sparse-signal model while effectively suppressing frequency aliasing [50]. 

However, it is crucial to recognize that compressive sensing does not fundamentally overcome the physical coherence limit imposed by the transverse relaxation time T_2_. The mathematical recovery guarantees of CS rely strictly on the a priori assumption of spectral sparsity within a parameterized dictionary. CS exploits structural sparsity to reconstruct signals, and it does not create new physical information. In practical FID magnetometers, deploying CS entails inherent trade-offs regarding estimator bias and robustness. Standard CS formulations project the continuous FID signal onto a discretized frequency dictionary. When the true Larmor frequency falls between discrete grid bins, this basis mismatch introduces spectral leakage and systematic estimation bias. Furthermore, the L_1_-norm regularization inherently penalizes large coefficients, causing amplitude and damping shrinkage that can bias parameter extraction. The robustness of CS is also challenged in operational environments where spatial magnetic field gradients or atomic spin-exchange dynamics introduce multi-exponential decay envelopes and time-varying phase perturbations. When the physical waveform deviates from the assumed idealized single-exponential damping model, CS reconstruction fidelity deteriorates rapidly, especially under low-SNR conditions near the detection threshold. 

Beyond estimation bias and robustness, the computational cost of CS presents a significant barrier to real-time closed-loop magnetometer operation. Compressive reconstruction relies on iterative convex optimization solvers, which impose substantial computational overhead and iterative latency. In contrast, established frequency estimators offer deterministic and highly efficient workloads suitable for embedded firmware implementations. For instance, nonlinear least squares (NLLS) estimation, such as the Levenberg–Marquardt algorithm, can continuously refine the Larmor frequency. Under appropriate signal and noise conditions, it can approach the theoretical CRLB without the shrinkage bias of L_1_-norm penalties. For ultra-low latency applications, traditional frequency counters estimate frequency by detecting zero-crossing events. They offer minimal computational complexity, but their precision degrades under low-SNR conditions [33]. To address this, Tong Gong et al. designed a high-sensitivity frequency counter specifically for FID signals, achieving a frequency sensitivity better than 100 μHz/√Hz at 10 Hz for a 200 Hz carrier signal [51]. To achieve high-bandwidth detection, Nathaniel Wilson et al. proposed a method based on instantaneous-phase retrieval. By tracking the phase evolution of the FID waveform in real time, this method enables measurement of arbitrarily modulated magnetic fields at frequencies of up to 50 times the Larmor frequency, substantially expanding the sensor’s bandwidth [52].

The reported developments indicate that the performance of an FID magnetometer is determined by the combined optimization of atomic polarization, coherence preservation, excitation control, optical readout, and signal estimation. Improvements in one subsystem may introduce new constraints in another. For example, increasing the vapor-cell volume can improve the signal amplitude but may increase power consumption and diffusion-related inhomogeneity, whereas miniaturization reduces the active volume and can increase wall-collision relaxation. Similarly, advanced signal-processing methods can improve frequency extraction from short FID waveforms, but their benefits depend on the validity of the assumed signal model, the signal-to-noise ratio, and the available computational resources. These cross-disciplinary trade-offs should be considered when comparing different FID magnetometer implementations.

## 5. Error Analysis and Environmental Adaptability

The measurement accuracy of OPMs is constrained by systematic errors. The pulsed operation of FID technology inherently provides advantages for suppressing such errors. The following discussion focuses on three aspects: the non-linear Zeeman effect and heading error suppression, dead-zone elimination, and co-magnetometer error correction, all aimed at enhancing measurement precision and environmental adaptability.

### 5.1. Non-Linear Zeeman Effect and Heading Error Suppression

The non-linear Zeeman (NLZ) effect originates from the non-linear splitting of atomic ground-state energy levels with magnetic field strength [53]. This effect causes FID signals to appear as superpositions of multiple frequency components, leading to linewidth broadening or shifts in the center frequency [54]. To compensate for the NLZ effect, D. P. Hewatt et al. proposed a dual-frequency fitting algorithm that adjusts hyperfine-level polarization weights, reducing magnetic field error to the 1 nT level [55]. Alternatively, microwave-driven Rabi frequency measurements can enable active NLZ compensation. Christopher Kiehl et al. used this approach in a vector magnetometer, achieving a vector sensitivity of 11 μrad/√Hz and an average vector accuracy of 0.46 mrad [56].

FID technology is also instrumental in suppressing heading errors in optically pumped magnetometers. Heading error is defined as the orientation-dependent difference between the estimated magnetic field and a reference value. The reported error may be expressed as peak-to-peak, RMS, maximum, or other metrics, and these definitions are not interchangeable. We therefore retain the convention used in each cited source and identify values with an unspecified metric accordingly.

Fundamentally, heading error represents orientation-dependent measurement discrepancies induced by changes in the relative geometry between the sensor frame and the ambient magnetic field vector. Rather than arising solely from asymmetric Zeeman sublevel populations, heading error is governed by a multifaceted coupling of physical mechanisms and signal estimation choices. Specifically, nonlinear and nuclear Zeeman effects split the single Larmor resonance into multiple unequally spaced transition lines. Under varying sensor-field orientations, the relative excitation amplitudes and phases of these unresolved hyperfine coherences shift systematically. When coupled with residual light shifts and estimator-dependent spectral phase pulling, this asymmetric multi-component superposition shifts the extracted central Larmor frequency. In pulsed and Bell–Bloom FID architectures, precise tailoring of optical modulation duty cycles, composite RF pulse sequences, and advanced multi-frequency fitting algorithms can effectively help balance sublevel transition weights and suppress multi-frequency interference. For example, S. Q. Liu et al. demonstrated that optimizing synchronous modulation parameters in a Bell–Bloom FID magnetometer suppresses the peak-to-peak heading error to below 1 nT across a wide angular range, as illustrated in Figure 9 [40].

### 5.2. Dead Zone Elimination Techniques

Detection dead zones represent a fundamental physical limitation of optically pumped magnetometers. They severely constrain performance in omnidirectional monitoring applications, such as satellite attitude control and underwater magnetic detection. In FID mode, when the measured magnetic field is parallel to the pump-beam direction, the system fails to generate a sufficiently large transverse spin component and thus enters a dead zone.

In conventional FID magnetometers, the signal amplitude depends strongly on the relative orientation between the measured magnetic field and the laser axis. As this angle approaches certain critical values, the FID amplitude drops below the detection threshold, leading to a substantial degradation in sensitivity or even complete signal loss [57]. Consequently, maintaining an appropriate sensor orientation is often necessary, which limits operational flexibility in practical applications. By applying short auxiliary magnetic field pulses along orthogonal directions, the atomic polarization vector can be forced to deviate from the axis of the currently measured field. Regardless of the direction of the external magnetic field, these auxiliary pulses ensure that a sufficient transverse precession component is induced. Liwei Jiang et al. utilized this approach in conjunction with the nonlinear magneto-optical rotation (NMOR) effect to eliminate directional blind spots, and to achieve full omnidirectional sensitivity coverage [58,59,60]. In addition to eliminating dead zones, pulsed magnetic-field excitation can facilitate multi-axis magnetic sensing. By carefully controlling the pulse sequence and timing, Shushan Gao et al. alternately acquired FID signals corresponding to different spatial directions, enabling vector magnetic-field measurements while maintaining high sensitivity [61].

In a single-beam rubidium-based FID system, Shrey Mehta et al. achieved dead-zone-free scalar detection by analyzing the characteristics of induced nonlinear coherent states during the free evolution stage, reaching a sensitivity of 3.2 pT/√Hz [57]. With this architecture, the system’s sensitivity to the magnetic field orientation is significantly reduced. The magnetometer can therefore maintain a stable Larmor frequency output even as the sensor rotates arbitrarily with its carrier. This advance is important for the development of quantum sensors deployed on handheld or mobile platforms.

### 5.3. Co-Magnetometer System Error Correction

Co-magnetometers utilize co-spatial coupling between alkali-metal and noble gas nuclear spins to suppress common-mode magnetic noise, enabling polarized detection of non-magnetic physical quantities such as inertial rotation. FID technology is a valuable tool for spin-state characterization and internal parameter calibration in these systems. Qi Yuan et al. used FID measurements of the longitudinal relaxation time and atomic transient response to characterize pump laser alignment in real time [62]. By combining time-domain FID responses with steady-state SERF monitoring, their system automatically adjusts mirror or laser angles to actively suppress alignment errors, significantly improving long-term stability [63].

The noble gas nuclear polarization level directly dictates the SNR of co-magnetometers. Tengyue Wang et al. measured ^129^Xe nuclear polarization via transverse and longitudinal in situ Rb magnetometry, using FID transient signals to detect the equivalent field of polarized nuclear spins with a sensitivity of ~370.4 fT/√Hz [64]. Zekun Wu et al. further enhanced transverse nuclear magnetization using an optimal oscillating field combined with FID detection, achieving a sensitivity of 500 fT/√Hz [65]. Morgan Hedges et al. proposed a co-magnetometry scheme based on controlled magnetic pulse perturbations and pulsed optical pumping, with the timing sequence illustrated in Figure 10. π/2 magnetic pulses alternately rotate the polarized noble gas around the *z*-axis. Optical pumping pulses re-polarize alkali atoms along z, and alkali precession between pulses encodes measurement information. By decoupling magnetic noise measurement rather than merely suppressing it, they achieved a magnetic-rotation crosstalk of 0.2 ± 0.1 μHz/pT in a Rb-Xe system [66].

## 6. Diverse Applications of FID Magnetometers

### 6.1. Biomagnetic Measurements

Biomagnetic measurements focus on the extremely weak magnetic fields generated by the electrophysiological activity of biological organisms. Compared with traditional SQUIDs, FID-based OPMs require no cryogenic cooling and can be positioned closer to the skin, thereby improving signal detection efficiency and spatial resolution [10,11].

The extraction of MEG and MCG signals represents a core challenge in biomagnetism [67]. Researchers have developed four-channel OPM arrays for MEG sensor systems [68]. The cited study explicitly reports operation in the FID mode and presents transient FID signals obtained after the spin-preparation interval. For MCG signal reconstruction, the BSVD algorithm has been applied to data acquired from pulsed FID magnetometers, enabling weak cardiac magnetic signals to be recovered from complex noise backgrounds [48]. In unshielded or semi-shielded clinical environments, ambient magnetic noise greatly exceeds biomagnetic signals. Gradiometric configurations comprising two or more FID sensing units are a potential approach to addressing this challenge. Differential processing cancels far-field common-mode environmental noise and isolates the near-field biomagnetic signal of interest [10,21]. In addition, optical multipass FID magnetometers increase the effective probe path length and improve the detection efficiency for weak magnetic signals, indicating their potential for biomagnetic measurements [32].

### 6.2. Geomagnetic Monitoring and Industrial Detection

FID OPMs exhibit excellent linearity and a wide dynamic range at the geomagnetic field scale, making them essential tools for geophysical exploration, spatial magnetic mapping, and industrial diagnostics.

Researchers have developed high-sensitivity magnetic sensing systems in the Bell–Bloom FID mode to monitor the urban magnetic environment, capturing low-frequency magnetic noise fluctuations with high precision for geomagnetic navigation and environmental research [8]. In broader geophysical applications, miniature commercial alkali-vapor FID magnetometers have been identified as promising alternatives or complements to fluxgate sensors in applications requiring small size, low power consumption, and high scalar sensitivity [7]. In the field of deep-space exploration, measurement accuracy is paramount. Yuefeng Lu et al. investigated a ^3^He-based FID magnetometer leveraging long nuclear-spin coherence times to achieve 100 ppb measurement accuracy in geomagnetic field environments, suitable as a magnetic reference for space missions [69]. Furthermore, FID magnetometers are increasingly utilized in industrial detection. High-resolution FID magnetic imaging technology is used to identify faults in integrated circuits (ICs) and to monitor lithium-ion batteries. Detecting the magnetic field images generated by internal current distributions enables non-contact diagnosis without damaging external casings [70]. Additionally, researchers have used rubidium FID-OPMs to characterize current noise in commercial constant current sources, providing calibration tools for high-performance electronic equipment [71].

With maturing integration technologies, FID-OPM-based sensors have transitioned into commercialized instruments. According to manufacturer specifications, the QuSpin QTFM Gen-2 shown in Figure 11a features a scalar sensitivity of 3 pT/√Hz [41], while the Twinleaf pulsed pump magnetometer (PPM) shown in Figure 11b specifies a sensitivity of 0.2 pT/√Hz above 10 Hz, representing a highly sensitive performance level among commercial Earth-field scalar magnetometers [42]. These products compress the sensor volume to the centimeter scale while maintaining pT-level sensitivity. They enable high-precision magnetic measurements via unmanned aerial vehicle (UAV) payloads or portable devices and eliminate the traditional reliance on bulky platforms and high-power driving circuits.

### 6.3. Fundamental Physics and Frontier Science

In fundamental physics research, FID magnetometers serve not only as ultra-sensitive magnetic field sensors but also as precision quantum probes for detecting non-magnetic interactions. Searching for dark matter and testing fundamental physical symmetries are highly challenging topics in modern physics. Certain dark matter candidates, such as axions or axion-like particles (ALPs), may couple with atomic spins to produce a “pseudo-magnetic field” effect. By constructing global networks of FID-based OPMs, researchers can collaboratively search for coherent spin precession signals induced by dark matter fields, thereby seeking evidence for ultra-light dark matter within Earth-scale “cavities”. In the Xiaodushan Desert, 120 km from Dunhuang City, Ariel Arza et al. conducted experiments using the QTFM Gen-2, validating the feasibility of FID magnetometers for ultra-light dark matter detection [72].

In experiments searching for the permanent electric dipole moment (EDM) of fundamental particles, which is crucial for understanding the matter–antimatter asymmetry of the universe, high-stability reference magnetometers are required to monitor environmental magnetic field drifts. M. Rosner et al. developed a high-stability FID-based magnetometer that serves as a high-precision reference probe, maintaining a drift of less than 50 fT over intervals ranging from 70 to 600 s. This provides a necessary experimental tool for investigating physical processes involving time-reversal symmetry breaking [73]. Furthermore, the transient evolution of FID signals provides an ideal experimental platform for observing complex quantum physical phenomena. In gradient magnetic fields, the evolution of diffusing atomic spins exhibits characteristics of non-Hermitian quantum mechanics. By precisely monitoring the envelope and frequency changes of FID signals, Xiangdong Zhang et al. successfully observed a phase transition phenomenon caused by parity–time (PT) symmetry breaking in an atomic vapor system [74]. This FID-based observation method provides an experimental pathway for exploring novel quantum phases and non-Hermitian topological physics in atomic systems.

The high sensitivity of FID magnetometers also enables application in microscopic biophysics. Magnetotactic bacteria are a special class of organisms capable of synthesizing nanoscale magnetic particles within their cells. In Figure 12, by utilizing high-sensitivity magnetometers, M. H. Ruiz et al. achieved real-time monitoring of the movement behavior and magnetic moment distribution of these bacterial populations under geomagnetic and perturbation fields [75]. The Pound–Drever–Hall (PDH) technique provides optical frequency locking and readout, whereas the reported operating sequence consists of pulsed spin preparation followed by detection of atomic free precession. Based on the operational definition adopted in this review, this sequence is classified as an FID measurement. This work not only contributes to the understanding of biological magnetotactic mechanisms but also provides a biological reference for the development of biomimetic magnetic sensors.

### 6.4. Magnetic Metrology and Calibration

OPM frequency outputs are directly traceable to atomic gyromagnetic ratios, which provide high accuracy and long-term stability for magnetic metrology. They are used for calibrating magnetic field generation equipment, measuring gradient tensors, and assessing material electromagnetic properties. Researchers have used RF-excited FID magnetometers for in situ coil calibration. By measuring the Larmor frequency at different current values, the coil constants are precisely determined and used to calibrate auxiliary sensors such as fluxgates, establishing a high-precision magnetic calibration system [33].

As illustrated in Figure 13, triaxial-coil non-orthogonality is a major source of error in integrated sensing systems. Dynamic FID response monitoring enables in situ calibration of both coil constants and geometric axis deviations, achieving full-parameter calibration of complex field-generation systems [76]. Multi-probe FID arrays enable magnetic gradiometers that precisely measure gradient tensors. By analyzing the rate of change of the magnetic field in different spatial directions, gradiometers can achieve precise localization of near-field magnetic dipoles. This gradient metrology technology has potential applications in underground obstacle detection and underwater defense [77]. As an emerging sensor architecture for electromagnetic induction (EMI) applications, FID atomic magnetometers can capture secondary fields from eddy currents. They show potential for mapping conductivity profiles and subsurface defects in industrial NDT and security screening [78].

## 7. Future Outlook and Challenges

Despite the remarkable progress achieved in recent years, several technical and engineering challenges remain before FID magnetometers can be widely deployed for practical applications. Future developments are expected to focus on chip-scale integration, robustness in unshielded environments, and the incorporation of advanced computational sensing techniques.

### 7.1. Fusion Bottlenecks in High-Level Integration

Advancing FID magnetometers toward chip-scale atomic magnetometer integration is pivotal for large-scale deployment. This process currently faces complex challenges arising from multi-physics field coupling [6]. First, miniaturization exacerbates wall-collision effects, which intensifies the loss of atomic coherence. This drives the research focus toward developing high-efficiency anti-relaxation coatings and optimizing buffer gas ratios to mitigate the conflict between miniaturization and sensitivity degradation [19,43]. Second, all-optical excitation schemes provide an attractive path toward miniaturization due to their simplified architecture and elimination of RF interference. However, they encounter severe engineering constraints during micro-integration. Achieving high-performance integration of VCSELs, precision optical components, and temperature control circuits within limited space is heavily restricted by thermal interference and stress coupling [25,30]. Furthermore, sustaining low-power operation while enhancing detection robustness in unshielded environments is crucial for the practical field deployment of chip-scale detectors. This requires the development of micro-scale non-inductive heating technologies and the integration of high-performance thin-film magnetic shielding.

### 7.2. Robustness in Complex Environments

Deploying FID magnetometers in unshielded, high-dynamic environments such as UAV surveys, industrial monitoring, and mobile healthcare requires overcoming several challenges. To address the “dead time” and bandwidth bottlenecks inherent in pulsed operation, future efforts may focus on developing quasi-continuous pulse schemes or instantaneous-phase retrieval techniques. These approaches balance high sampling rates with frequency-discriminating resolution [52]. In unshielded environments with strong field gradients, reducing the vapor cell volume and constructing differential gradiometer architectures are key pathways for suppressing common-mode noise while preserving atomic coherence [21]. Additionally, the introduction of intelligent phase-locked loops (PLLs) and adaptive pulse sequence adjustment algorithms can effectively enhance dynamic tracking in the presence of sharp background field fluctuations or carrier rotation. This ensures robust real-time monitoring across a broad dynamic range.

### 7.3. Intelligent Signal Processing and Sensing

Looking forward, machine learning and physics-informed neural networks (PINNs) may provide additional tools for feature extraction and parameter estimation in FID magnetometers, including the modeling of systematic errors. However, their practical deployment on low-power edge platforms remains challenging and has not yet been fully demonstrated for FID sensing. Complex PINN models may require substantial computational resources and introduce additional inference latency, which can conflict with the real-time operation and power constraints of portable sensors. Possible strategies include reduced-order models, integer quantization, structural pruning, and hybrid architectures that combine fast deterministic frequency estimators with lower-rate neural corrections. The practical benefits of PINNs should therefore be assessed according to the requirements of specific applications, particularly in terms of accuracy, computational cost, and robustness under varying magnetic-field conditions.

## 8. Conclusions

This paper systematically reviews the technical evolution of FID optically pumped magnetometers, tracing their development from laboratory phenomena to core quantum sensing tools that combine high sensitivity and high accuracy. By virtue of the temporal isolation between pumping and evolution, the FID mode reduces the influence of systematic effects, such as light shifts. Advances in MEMS fabrication and optical multipass enhancement have improved the SNR of miniaturized FID magnetometers, while signal demodulation methods such as BSVD and instantaneous phase retrieval have expanded the range of signals from which parameters can be extracted. This progress spans applications from biomagnetic imaging to geomagnetic monitoring and fundamental physics. In the future, advances in system integration, robustness in unshielded environments, and quantum-enhanced sensing may further establish FID magnetometers as versatile quantum measurement tools for precision sensing applications.

## Figures and Tables

**Figure 1:**
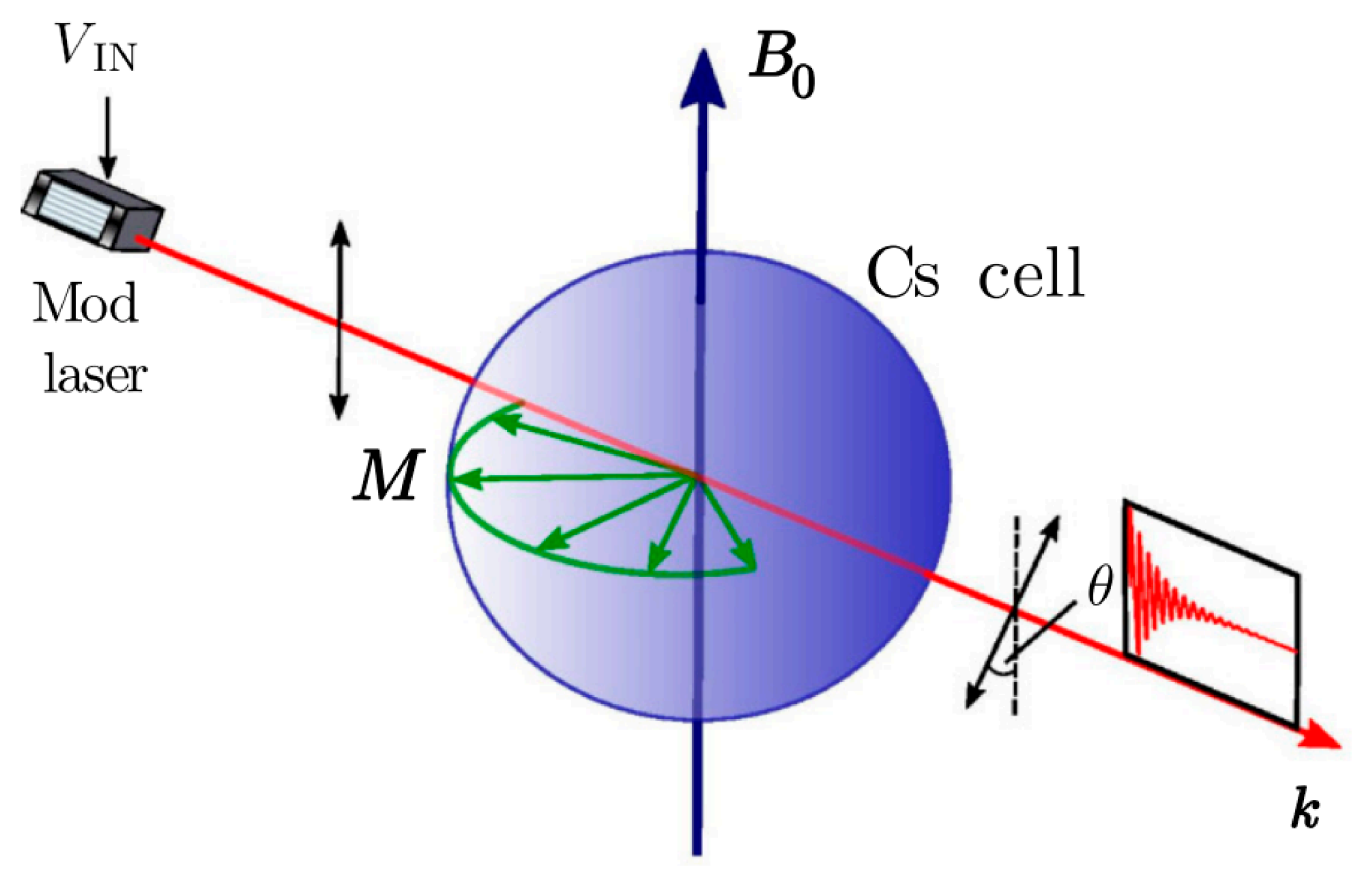
Schematic illustration of the basic operating principle of an FID optically pumped magnetometer. A modulated laser beam is directed into the cesium vapor cell to optically pump the atomic ensemble and to establish a polarized magnetization vector ***M***. After the preparation or tipping process, the optical driving field is either removed or sufficiently detuned. The atomic magnetization then undergoes free precession around the ambient magnetic field *B_0_*. The precessing transverse magnetization changes the polarization state of the transmitted light, producing a damped time-domain FID waveform. The oscillation frequency of this waveform is used to determine the magnitude of the magnetic field; *θ* denotes the optical polarization-rotation angle and *k* indicates the propagation direction of the optical beam [19].

**Figure 2:**
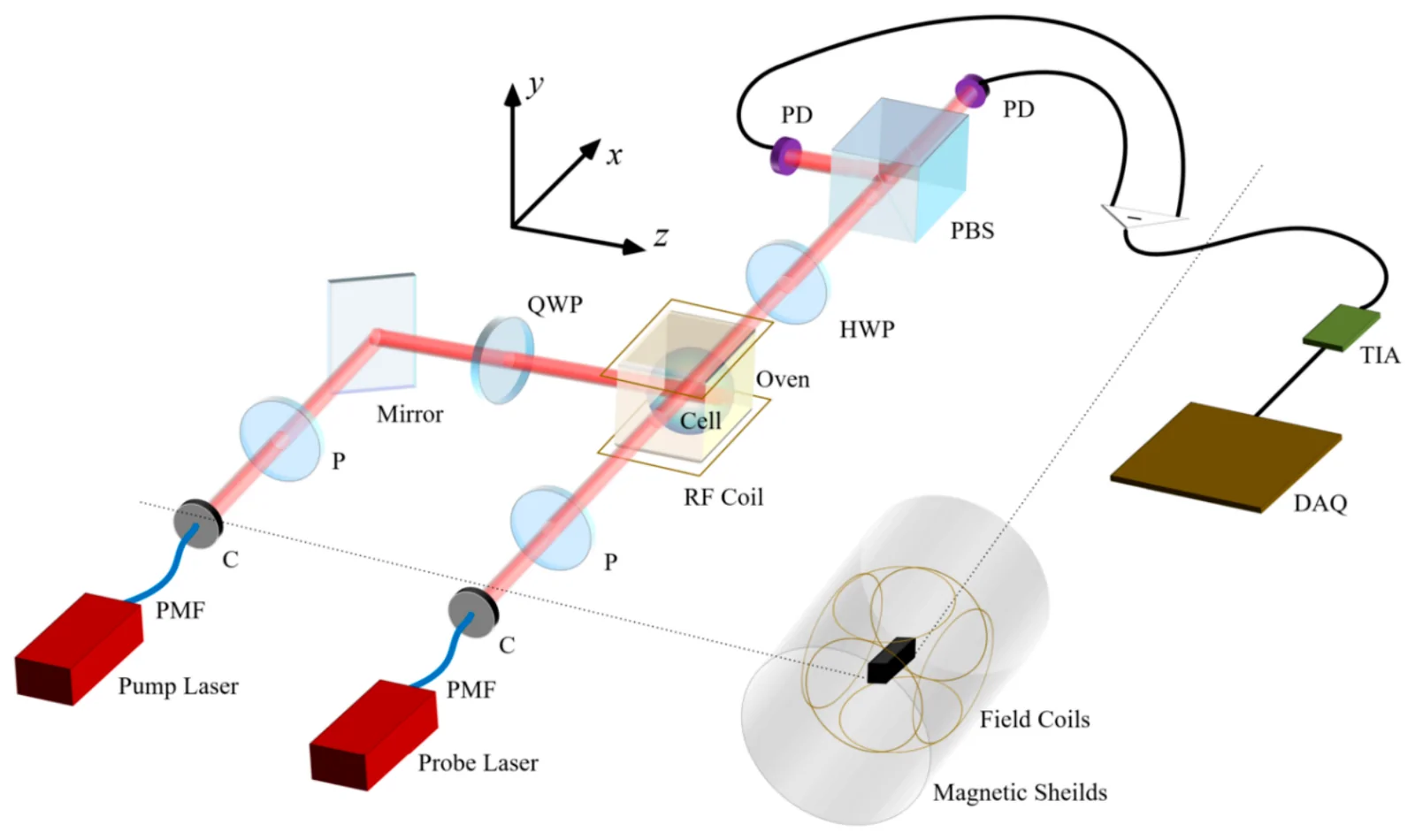
Schematic of a synchronous-optical-pumping FID magnetometer with RF pulse modulation [20]. Laboratory and magnetic-field reference frames are shown, with the pump, probe, and RF fields directed along the *z*, *x*, and *y*-axes, respectively. Synchronous optical pumping prepares the spin polarization. A transverse RF pulse tips the polarization into the plane perpendicular to B_0_. The atomic ensemble then freely precesses around B_0_ during the probe stage, generating the FID signal.

**Figure 3:**
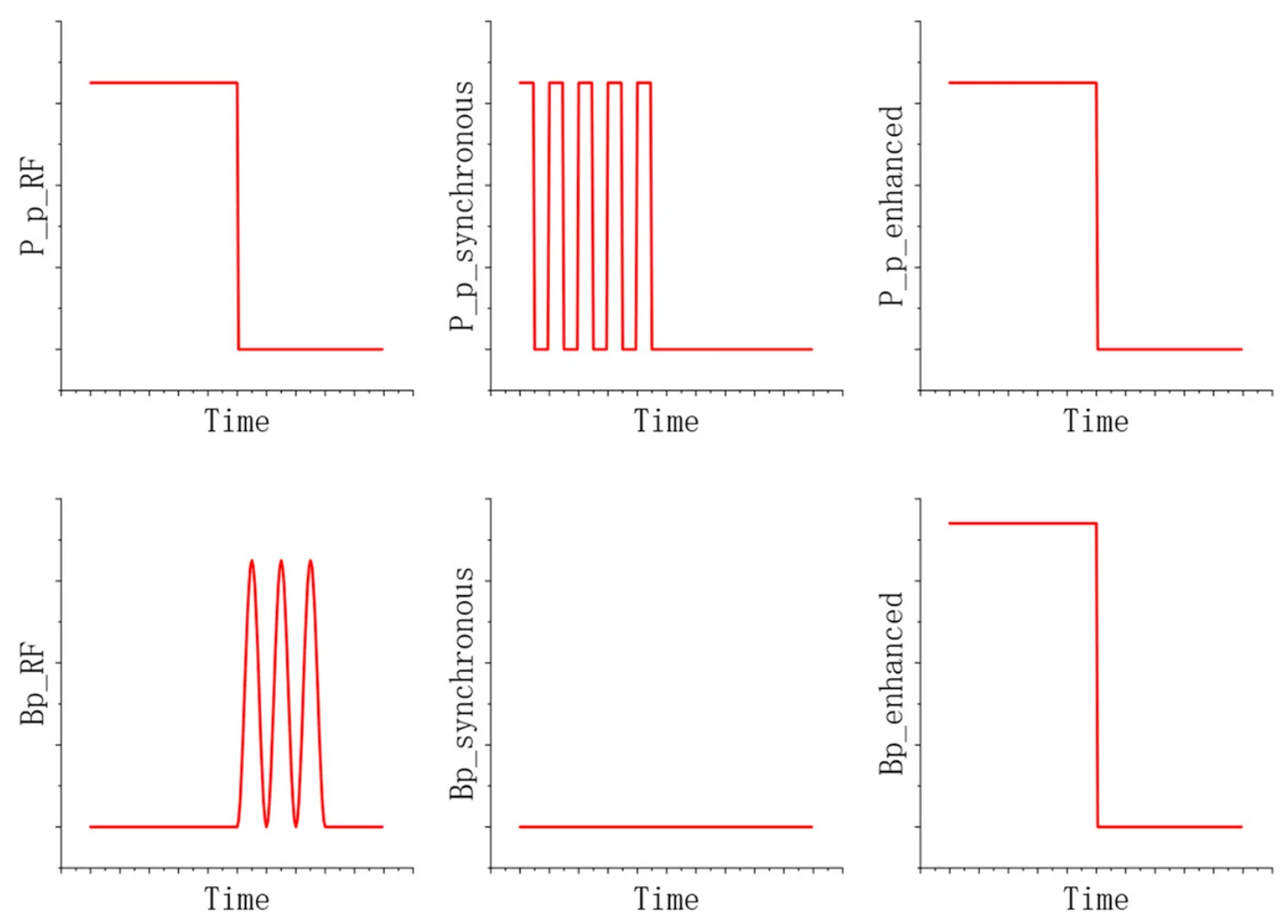
Timing diagrams of three FID excitation architectures: RF excitation (**left**), light-modulated optical pumping (**center**), and magnetic-field-enhanced optical pumping (**right**). The diagrams compare the temporal application of optical pumping and RF or auxiliary magnetic-field excitation during spin preparation, followed by the removal of the driving fields for FID free precession.

**Figure 4:**
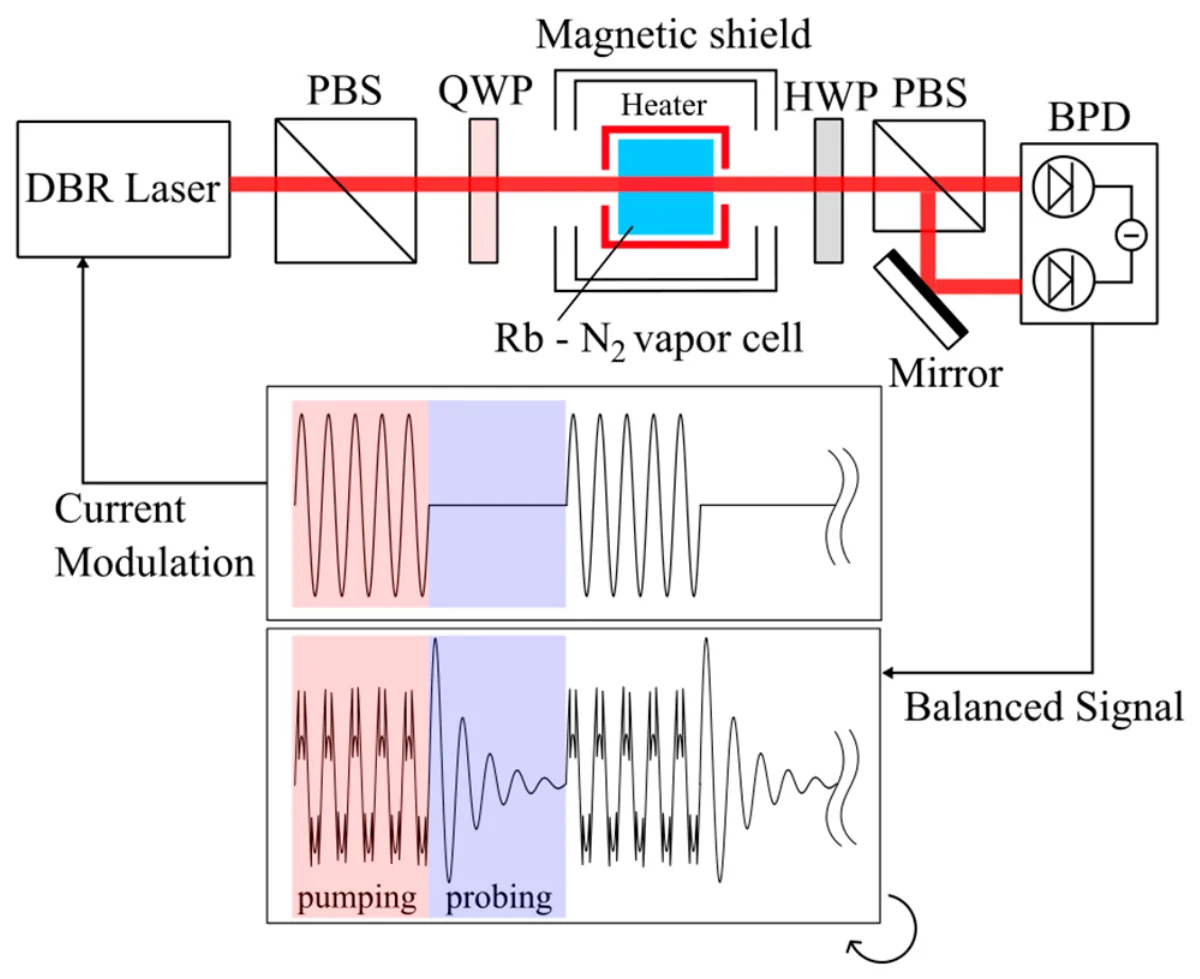
Experimental setup of a single-beam all-optical pulsed FID magnetometer based on distributed Bragg reflector (DBR) laser mode hopping [30]. A burst sine-wave modulation of the laser injection current produces pulsed pumping by switching the laser frequency across the mode-hop point. The same beam is used for optical pumping (red region) and probing (blue region); after the Rb–N_2_ vapor cell, the Faraday-rotation signal is detected with a balanced polarimeter.

**Figure 5:**
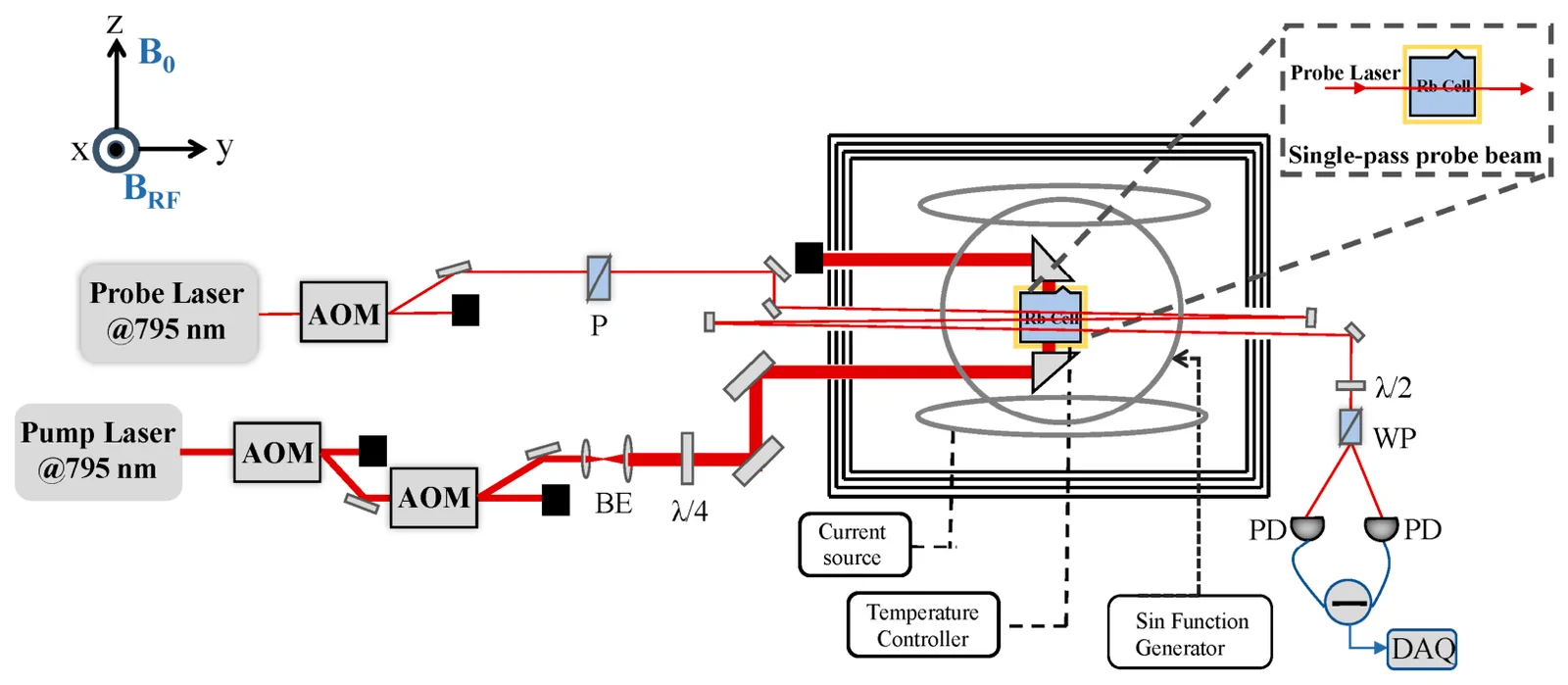
Schematic of an RF-excited FID rubidium magnetometer with a multipass probe path [32]. Separate pump and probe lasers prepare and interrogate the vapor cell, respectively. The pump beam is circularly polarized, whereas the probe beam traverses the cell multiple times between high-reflectivity mirrors. A transverse RF field tips the atomic polarization, and the resulting optical-rotation signal is detected by a balanced polarimeter and recorded by a data acquisition system.

**Figure 6:**
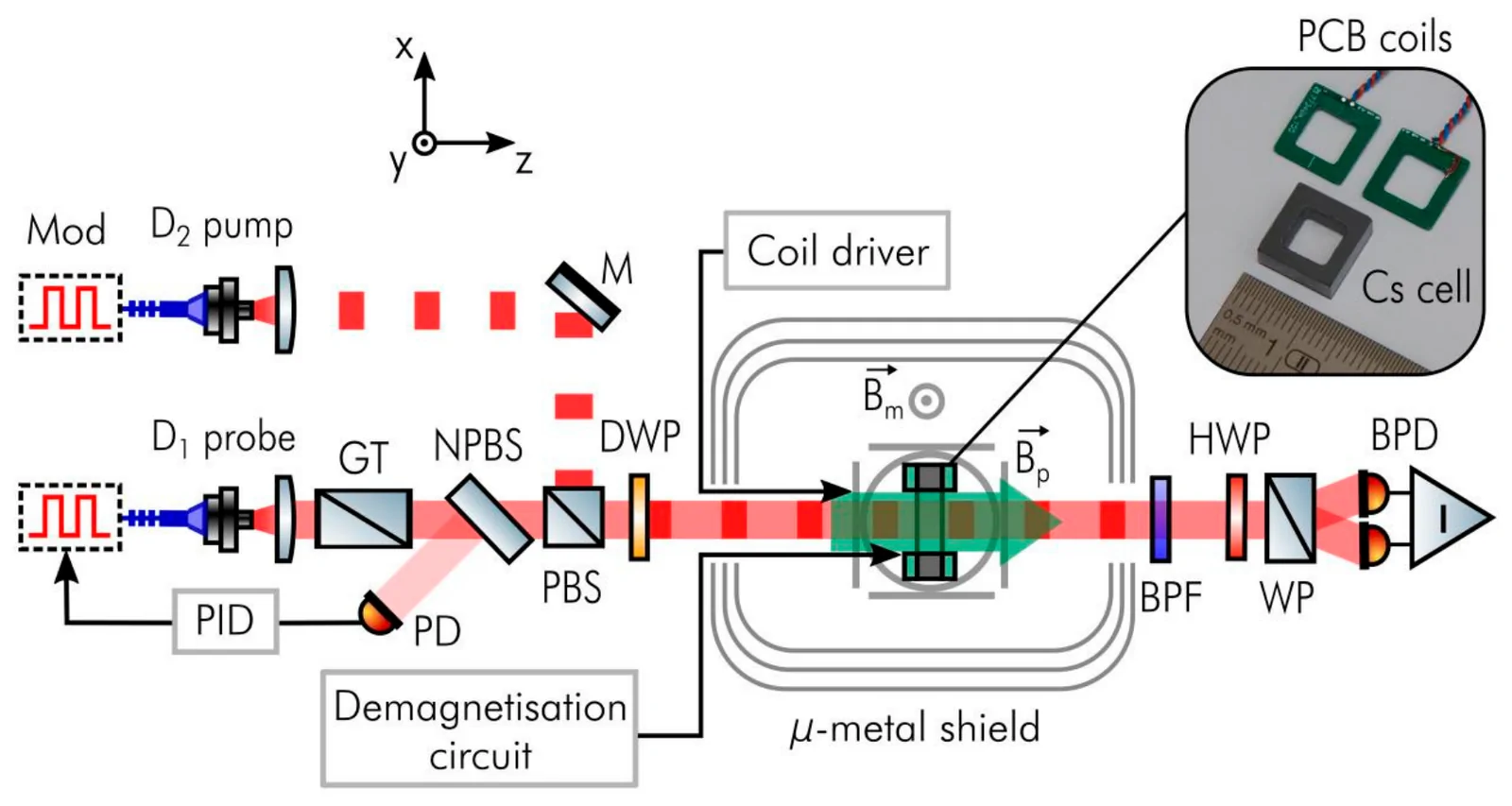
Schematic of a two-beam FID magnetometer using magnetic-field-enhanced optical pumping [36]. Co-propagating D_2_ pump and D_1_ probe beams interact with the Cs vapor cell inside a shield. PCB coils provide a longitudinal auxiliary field B_p_ during pumping and are rapidly demagnetized before readout. The probe polarization rotation is then analyzed using a Wollaston prism and a balanced photodetector.

**Figure 7:**
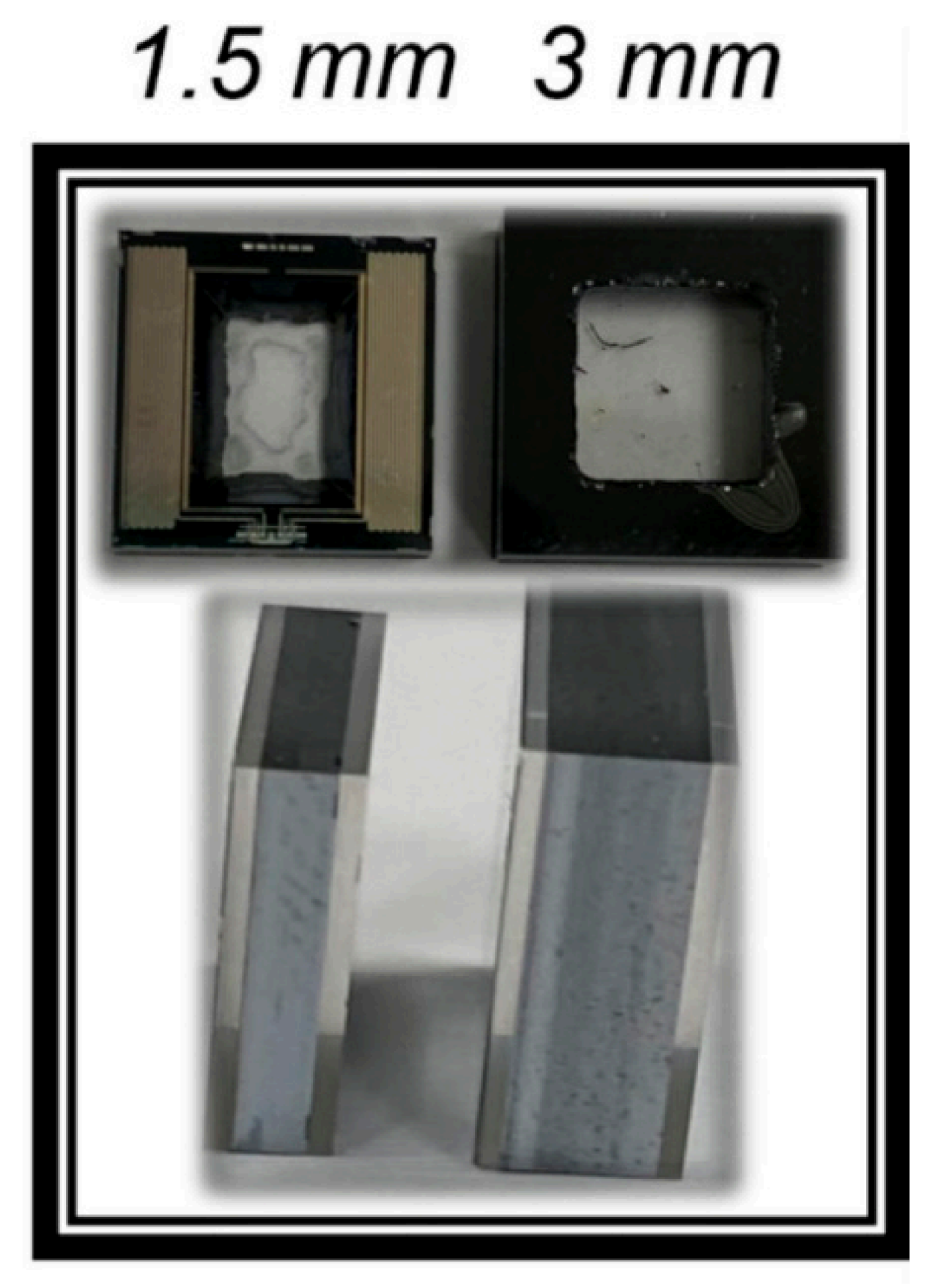
Photographs of the 1.5 and 3 mm thick cesium MEMS vapor cells used for FID magnetometry [37]. The top and side views highlight the compact cell geometry and the thickness variations investigated to balance miniaturization, atomic coherence, and signal strength.

**Figure 8:**
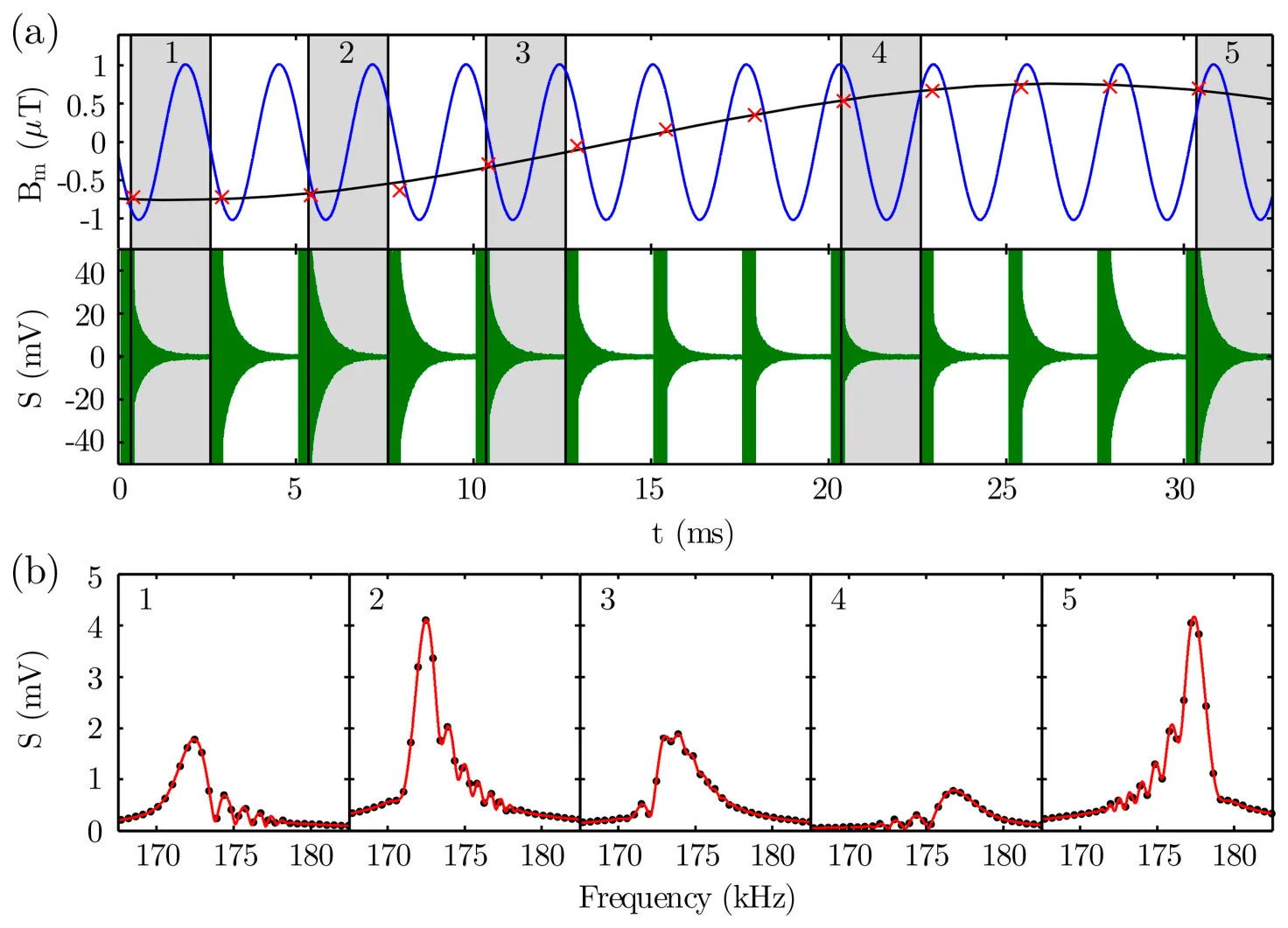
Schematic of signal reconstruction [49]. (**a**) Top: oscillating component of the magnetic field (blue line) generated by the modulation coils, aliased reconstructed waveform (red crosses), and corresponding fit to a sinusoidal model (black line). Bottom: time series of an FID signal train (green trace). (**b**) Snapshots of frequency-modulated FID spectra (black dots) and their associated best fits converted to the frequency domain (red lines). The numbers (top left) refer to the corresponding time-domain signals, which are highlighted by a grey background.

**Figure 9:**
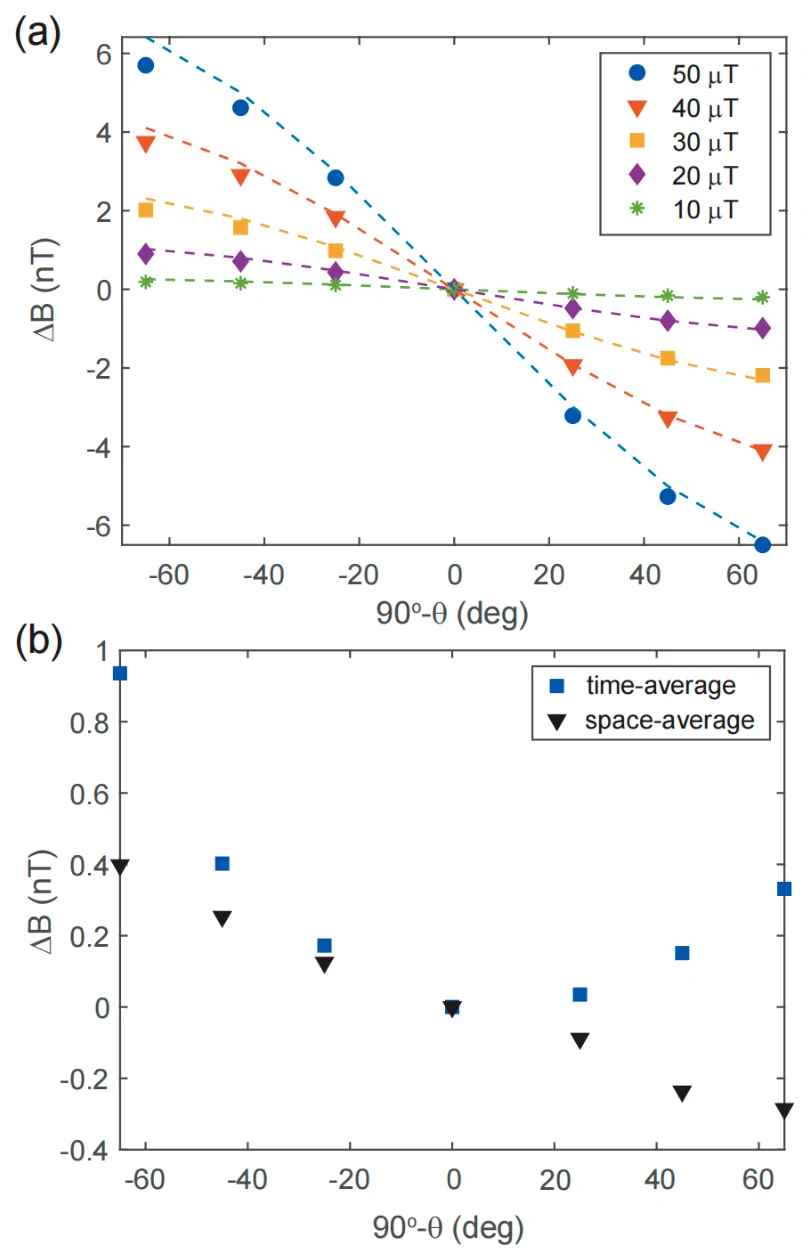
(**a**) Theoretical functional relationship between the heading error and the angle θ. (**b**) Peak-to-peak heading error after suppression [40].

**Figure 10:**
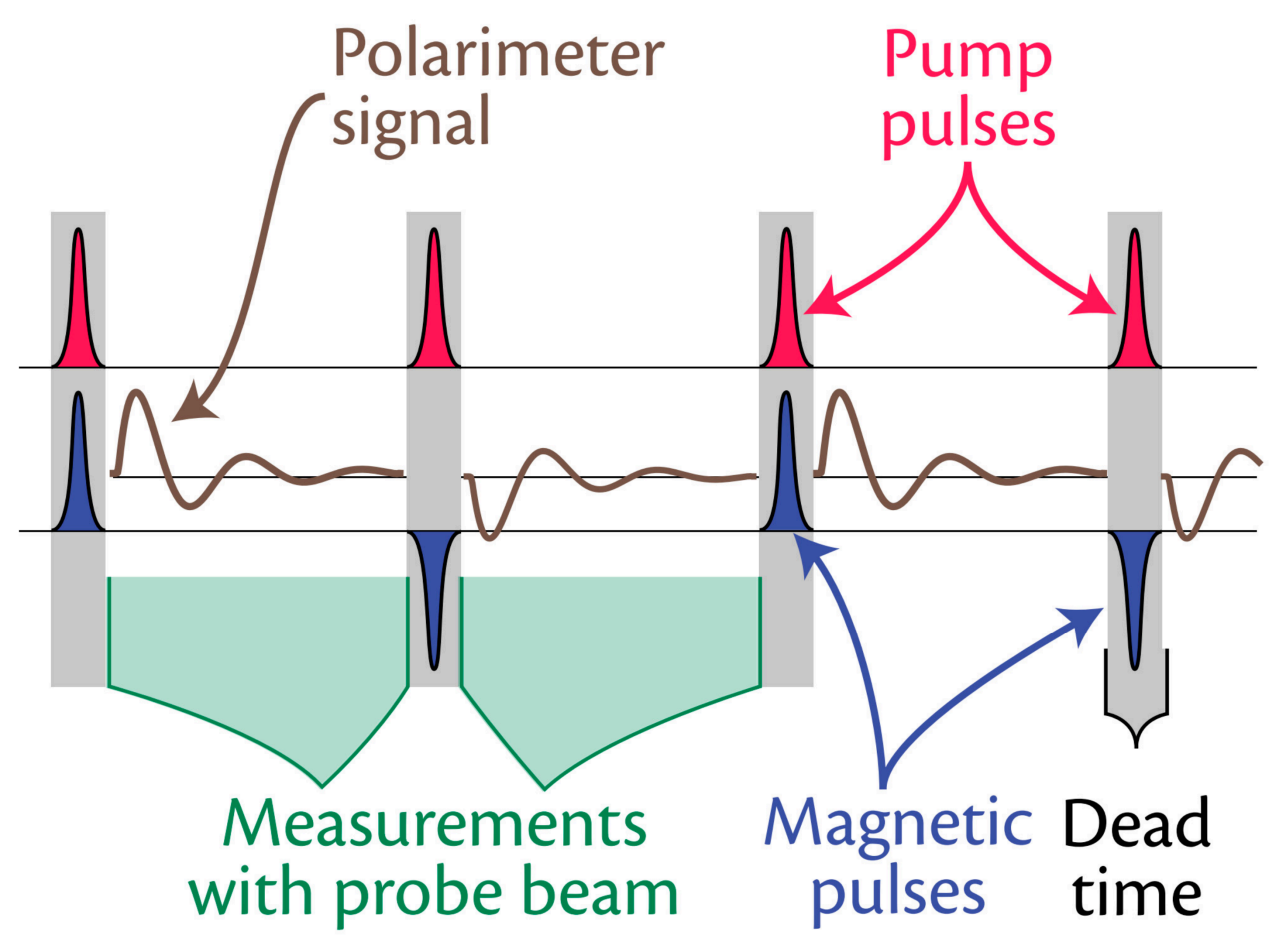
Pulse sequence employed in a co-magnetometer [66].

**Figure 11:**
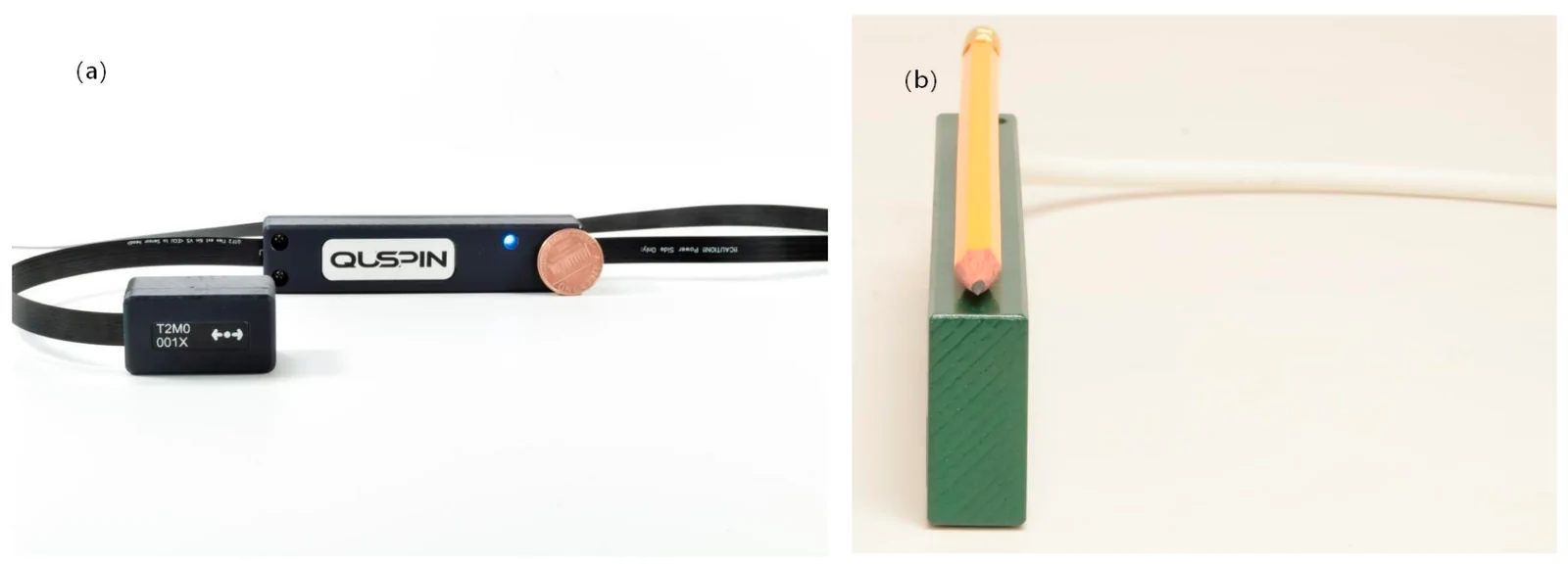
(**a**) QuSpin QTFM Gen-2 [41]. (**b**) Twinleaf PPM [42].

**Figure 12:**
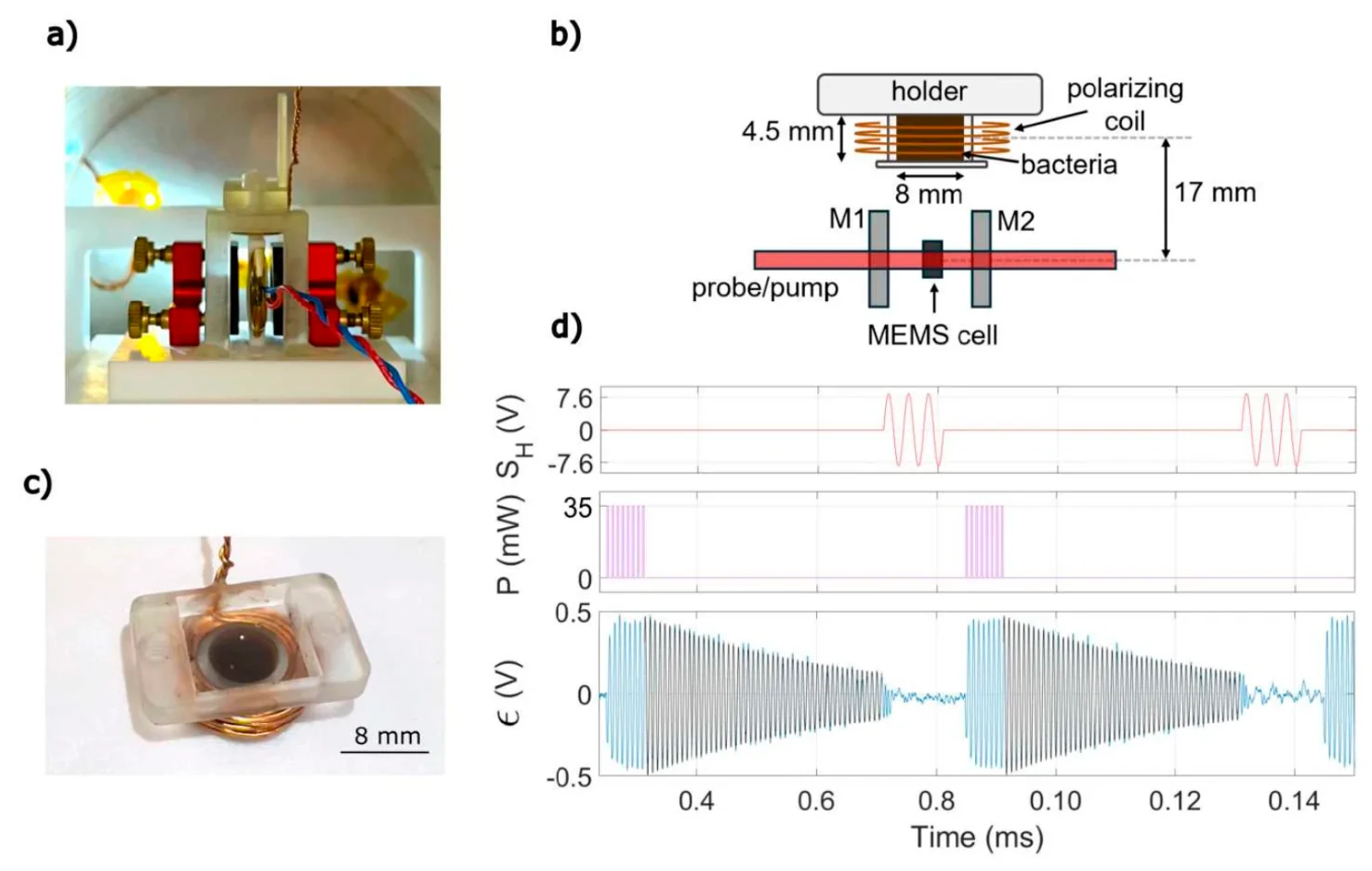
Magnetotactic bacterial populations studied. (**a**) Picture of the optical cavity in the shield with one cap open. (**b**) Schematic of the sample holder design, polarizing coil, and cavity setup. (**c**) Picture of the opened bacteria holder containing the solution. (**d**) Top: Voltage sent to the heater SH, shown in red. Middle: Pump power sent before the cavity, shown in purple. Bottom: Error signal ϵ following atomic precession when locked to the zero-crossing point. Blue line represents raw data and black line is a fitted curve [75].

**Figure 13:**
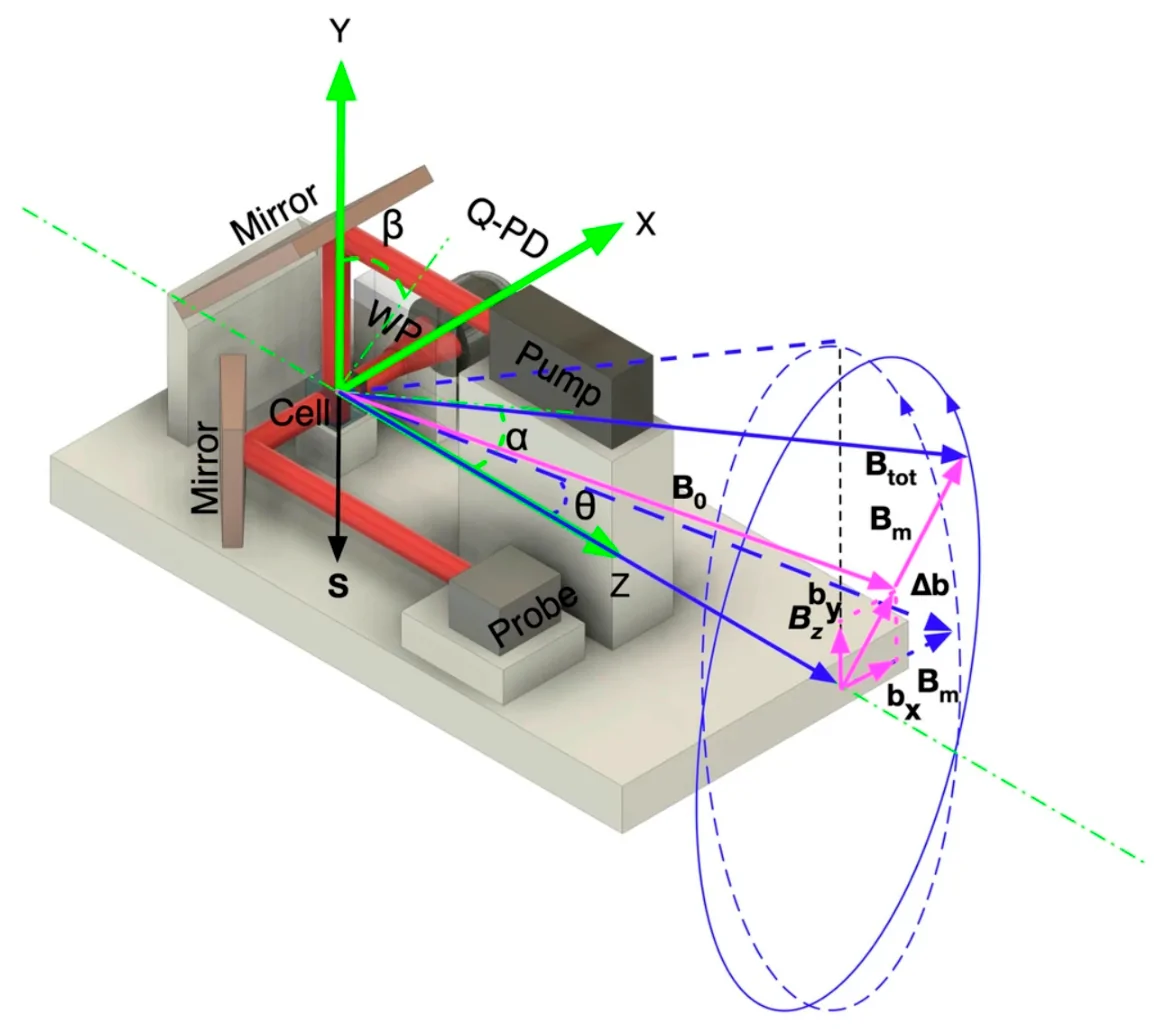
Pulsed vector atomic magnetometer using an alternating fast-rotating field. Experimental setup: Three coils define the x, y, and z directions [77].

**Table 1:** Comparative summary of representative FID optical-pumping magnetometer architectures and reported performance.

Work	Scheme	Field Regimen	Cell Dimensions	Sensitivity	Reported Bandwidth	Cycle Rate	Accuracy	Heading Error	Dead Zones
Liu et al. (2025) [34]	RF	Total Field	spherical vapor cell with a 10 mm diameter	0.47 pT/√Hz	250 Hz	N/A	Below 4.5 nT	N/A	N/A
McWilliam et al. (2023) [37]	Light-Modulated	Total Field	1.5 mm thick MEMS cell	2.5 pT/√Hz	500 Hz	1000 Hz	N/A	N/A	N/A
Li et al. (2022) [38]	RF	Total Field	cylindrical glass cell, d = 25 mm and a length of L = 50 mm.	0.7 pT/√Hz	1000 Hz	2000 Hz	N/A	N/A	N/A
Gerginov et al. (2020) [39]	Light-Modulated	Low Field (<10 μT)	3 × 3 × 2 mm^3^ MEMS cell	sub 0.1 pT/√Hz	500 Hz	N/A	N/A	N/A	along the optical axis
Liu et al. (2025) [40]	Light-Modulated	Total Field	ϕ12.7 × 11.5 mm^3^ Herriott cavity	N/A	31.25 Hz	N/A	N/A	Below 1 nT	±25° along the pumping optical axis
QuSpin QTFM Gen-2 [41]	Magnetic Field-Enhanced	Total Field	N/A	3 pT/√Hz	500 Hz	1000 Hz	N/A	Below 3 nT	±7° along the optical axis
Twinleaf PPM [42]	Light-Modulated	Total Field	N/A	0.2 pT/√Hz	N/A	500 Hz	N/A	±5 nT	±7° along the optical axis

Note: Bandwidth and cycle rate represent different performance metrics: bandwidth characterizes a reported spectral, estimator, or tracking response, whereas cycle rate denotes the repetition frequency of the FID measurement sequence. Heading error values are reported using the metric specified in the cited source, such as peak-to-peak, RMS, maximum, or range. Values with different reporting conventions should not be directly compared.

## Data Availability

This article is a review of published literature. No new data were created or analyzed in this study. All relevant information is contained within the manuscript and its cited references.

## Abbreviations

The following abbreviations are used in this manuscript:

FID	Free Induction Decay
MEMS	Micro-Electro-Mechanical Systems
OPMs	Optically Pumped Magnetometers
SQUIDs	Superconducting Quantum Interference Devices
MEG	Magnetoencephalography
MCG	Magnetocardiography
MAD	Magnetic Anomaly Detection
CW	Continuous-Wave
RF	Radio-Frequency
SVD	Singular Value Decomposition
NDT	Non-Destructive Testing
PBS	Polarizing Beam Splitter
SNR	Signal-to-Noise Ratio
SERF	Spin-Exchange Relaxation-Free
EMI	Electromagnetic Interference
AM	Amplitude Modulation
FM	Frequency Modulation
MPC	Multipass Cell
FFT	Fast Fourier Transform
BSVD	Backward Singular Value Decomposition
CS	Compressive Sensing
NLZ	Nonlinear Zeeman
NMOR	Nonlinear Magneto-Optical Rotation
ICs	Integrated Circuits
ALPs	Axion-Like Particles
EDM	Electric Dipole Moment
PT	Parity–Time
AI	Artificial Intelligence
CNNs	Convolutional Neural Networks
RNNs	Recurrent Neural Networks
PINNs	Physics-Informed Neural Networks
PLLs	Phase-Locked Loops
UAV	Unmanned Aerial Vehicle
